# Manipulation and Malicious Personalization: Exploring the Self-Disclosure Biases Exploited by Deceptive Attackers on Social Media

**DOI:** 10.3389/frai.2019.00026

**Published:** 2019-11-29

**Authors:** Esma Aïmeur, Nicolás Díaz Ferreyra, Hicham Hage

**Affiliations:** ^1^Department of Computer Science and Operations Research (DIRO), University of Montreal, Montreal, QC, Canada; ^2^Research Training Group “User-Centred Social Media” University of Duisburg-Essen, Duisburg, Germany; ^3^Computer Science Department, Notre Dame University-Louaize, Zouk Mosbeh, Lebanon

**Keywords:** adaptive privacy, awareness, malicious personalization, self-disclosure, cognitive biases, deception, social media

## Abstract

In the real world, the disclosure of private information to others often occurs after a trustworthy relationship has been established. Conversely, users of Social Network Sites (SNSs) like Facebook or Instagram often disclose large amounts of personal information prematurely to individuals which are not necessarily trustworthy. Such a low privacy-preserving behavior is often exploited by deceptive attackers with harmful intentions. Basically, deceivers approach their victims in online communities using incentives that motivate them to share their private information, and ultimately, their credentials. Since motivations, such as financial or social gain vary from individual to individual, deceivers must wisely choose their incentive strategy to mislead the users. Consequently, attacks are crafted to each victim based on their particular information-sharing motivations. This work analyses, through an online survey, those motivations and cognitive biases which are frequently exploited by deceptive attackers in SNSs. We propose thereafter some countermeasures for each of these biases to provide personalized privacy protection against deceivers.

## 1. Introduction

Nowadays, Social Network Sites (SNSs) like Facebook, Instagram, or Snapchat are widely used for connecting with friends, acquaintances, or even meeting new people. Basically, these sites have become regular meeting places and redefined, to a large extent, the way people create and maintain social relationships (Joinson, 2008; Penni, 2017). Mainly, SNSs allow people to interact simultaneously with a vast network of users and, thereby, maximize their “social capital.” Like in the real world, social links in SNSs are reinforced by disclosing more personal information to others. However, the volume and type of content shared online is larger and more diverse than the one revealed offline (Stutzman et al., 2011; Such and Criado, 2018). Moreover, the time people spend sharing information in SNSs has exponentially increased over the last years (Smith and Anderson, 2018). In consequence, SNSs are appealing to individuals with harmful intentions who see these virtual spaces as valuable sources of private information.

In SNSs, privacy as a human practice acquires a high importance since these are spaces in which users make their private life public. That is, users voluntarily disclose their private information to wide and—sometimes untrusted—audiences through the different communication channels available in these platforms (e.g., instant messaging, posts, stories) (Acquisti and Gross, 2006; Boyd, 2010). However, although users in general have reported high concerns about their privacy, they tend to disclose personal information without foreseeing the potential negative effects. Moreover, they often relay on lax privacy settings and consider their online peers as trusted, which increases significantly the chances of being victims of a malicious user. Consequently, users often regret having shared their personal information in SNSs after they suffer unwanted incidents like *cyber-bullying, reputation damage*, or *identity theft* (Wang et al., 2011).

Currently, cyber-attacks tend to focus more on human vulnerabilities instead of flaws in software or hardware (Krombholz et al., 2015). For instance, about 3% of Malware attacks exploit technical lapses while the other 97% target the users through social engineering^1^. In order to gain trust and manipulate their victims, social engineers often employ *online deception* as their attack vector (Tsikerdekis and Zeadally, 2014; Krombholz et al., 2015). Particularly, *deceivers* hide their harmful intentions and mislead other users to reveal their credentials (i.e., accounts and passwords) or perform hazardous actions (e.g., install Malware) (Aïmeur and Sahnoune, 2019). For instance, they often impersonate trustworthy entities using fake SNSs accounts to instigate other users on accessing insecure web links and install malicious software. For this, deceivers exploit users' *motivations*, such as financial or moral gain, and employ different *incentive strategies* to mislead them, accordingly (Albladi and Weir, 2016). Such strategies can take the form of a fake link to a cash prize, or a fake survey on behalf of a prominent non-profit organization.

Understanding the users' motivations is fundamental for the design and success of incentive mechanisms. Particularly, motivations have been widely studied and leveraged to increase users' participation in social applications like discussion forums or web blogs (Vassileva, 2012). As a result, several guidelines and patterns have been elaborated on how to design social interfaces that can attract and sustain active contributions in these virtual communities. However, similar principles can be employed in the design of deceptive strategies that mislead users to reveal personal information. Moreover, as in social applications, these incentives can be personalized to each user (victim) to maximize their effect (damage). This process, in which deceivers use the motivations and cognitive biases of their victims to craft their attacks, can be considered as a case of *malicious personalization* (Conti and Sobiesk, 2009).

This work investigates those motivations and cognitive biases that can be exploited for malicious personalization in SNSs. Particularly, it examines which are the self-disclosure motivations and biases that can be leveraged by deceivers to mislead users into revealing private information. Furthermore, this paper analyses (i) which are the incentive strategies used by deceivers in their attacks, and (ii) the link between self-disclosure motivations and specific categories of personal information. To better understand the role that self-disclosure biases (i.e., cognitive and motivational) have in deceptive attacks, we conducted an online survey with 349 participants via Amazon Mechanical Turk (Mturk). Based on our findings, we elaborate on countermeasures oriented to provide personalized privacy protection against deceivers. In particular, we underline how the findings of this work contribute to the development of personalized risk awareness mechanisms.

The rest of the paper is organized as follows. In the next section, related work on online deception is discussed and analyzed. Following, section 3 introduces the theoretical foundations of this paper. Particularly, the use of motivations and incentives for the design of persuasive technologies is discussed together with role of self-disclosure biases in malicious personalization. Sections 4 and 5 elaborate on the design of our online survey and its results, respectively. Next, in section 6, deception countermeasures based on adaptive risk awareness are elaborated, and the limitations of our approach are discussed. Finally, in section 7, we outline the conclusions of this paper and introduce directions for future work.

## 2. Related Work

Analysing and understanding the logic behind cyber-attacks is fundamental for developing security and data protection countermeasures. Unlike attacks that focus solely on technical vulnerabilities, social engineering attacks target users with access to critical information. That is, they mislead people into disclosing confidential information or even carrying out hazardous actions through influence and persuasion. There are several types of social engineering attacks each of them relying on different technical, physical and social assumptions. Krombholz et al. (2015) analyzed closely a number of well-known and advanced social engineering attacks like phishing, waterholing and baiting, to determine which are their respective underlying assumptions. As a result, they introduced a taxonomy which classifies these attacks according to (i) the communication channel they exploit (e.g., e-mail, cloud, website), (ii) the operator of the attack (i.e., a human or software), and (iii) the strategy they use to approach the victim (i.e., physical, technical or socio-technical). In line with this approach, Aïmeur et al. (2018) introduced a taxonomy which classifies deceptive attacks in SNSs according to their strategy (i.e., *information harvesting, social influence*, or *identity deception*). Such a taxonomy also prescribes a set of preventative strategies for each attack category based on state-of-the-art technologies.

As mentioned in section 1, online deception occurs when social engineers employ manipulation and persuasion techniques to mislead their victims. Hence, the success of a deceptive attack will depend, to a certain extent, on the victim's attitude toward manipulation, their risky behavior and their trust in the perpetrator. Such factors were analyzed by Aïmeur and Sahnoune (2019) in the context of online relationships through a survey-based experiment. Among other findings, the study revealed that users who have been involved in an online relationship are more likely to give away their private information when asked for it. Further research has focused on methods for detecting fake identities in SNSs (Alowibdi et al., 2015; van der Walt and Eloff, 2017). Particularly, on using behavioral indicators (e.g., absence of profile picture or suspicious online activity) to identify those accounts that may be administrated by deceivers. However, to the best of our knowledge, not much effort has been made on understanding the self-disclosure biases that are exploited by deceivers to craft their attacks. Consequently, this work investigates the effect of these biases under various deceptive scenarios. Particularly, we analyse the role of incentives and motivations when people self-disclose as the value they assign to particular pieces of private information.

## 3. Theoretical Background

Following, the theoretical foundations of this work are introduced. Particularly, we discuss the most relevant perspectives on motivation that exist in the literature and their role in the context of deceptive attacks. In line with this, we examine the different self-disclosure motivations and incentive mechanisms that can be leveraged for the elaboration of such attacks. The concepts introduced in this section set the basis for the elaboration of our online survey.

### 3.1. Motivations and Incentives

Understanding the motivations behind human behavior has guided, to a large extent, the research agenda of disciplines like economics and psychology (Kraut and Resnick, 2012). Each of these disciplines address the issue of motivation under different assumptions related to the rationality of peoples' decisions and the environment in which such decisions are taken. For instance, classical economics considers people as rational agents that interact in an environment in which certain behavior has associated a particular pay-off (positive or negative) (Vassileva, 2012). In this case, incentive mechanisms are designed to ensure that the overall community fulfills a particular goal (e.g., optimizing the joint welfare of all the individuals) without taking into account the diversity of motivations among its members. Hence, this approach emphasizes the benefit of the community as a whole rather than the one of its members.

Behavioral economics, on the other hand, considers people as irrational and investigates the social, cognitive and emotional factors that may influence their actions. Particularly, this approach has shown that many classical mechanisms are not psychologically valid, and therefore fail on explaining the reasons behind peoples' actions, willingness, and goals (Ariely, 2008; Vassileva, 2012). Furthermore, contributions in the area of behavioral economics have nourished principles of user engagement in the design of information systems. One of the most prominent ones is the incorporation of “gamification” elements (e.g., motivational patterns, rules and feedback loops) in social computing applications to increase users' participation (Hamari and Koivisto, 2013). The use of gamification elements is often grounded in psychological theories, such as the *reinforcement theory* (Skinner, 1969) and the *expectancy theory* (Vroom, 1964), which emphasize the influence of external rewards on people's behavior.

Although gamification has been widely explored in the design of social computing applications, it is often questioned because it relies solely on the use of rewards to generate a motivational effect on users. That is, it often overlooks the effect that intrinsic motivations like enjoyment or personal values may have in peoples' behavior (Vassileva, 2012). Moreover, it also neglects the relevance of motivational factors coming from peoples' social environment, such as status and recognition. Consequently, a considerable amount of research focus on developing motivational strategies that elaborate on such intrinsic and social factors (Ling et al., 2005; Burke et al., 2009; Kraut and Resnick, 2012; Chang et al., 2016). Furthermore, approaches on the personalization of incentives have also been introduced to increase users' participation and engagement in social applications (Berkovsky et al., 2012). The main premise of personalized incentives is that motivations are always personal and vary from individual to individual. Consequently, adapting the incentives and rewards to each particular user can enhance significantly the effectiveness of a motivational strategy (Masthoff et al., 2014).

### 3.2. Self-Disclosure Biases

As mentioned in section 1, deceivers exploit cognitive and motivational biases that contribute to online self-disclosure to shape their attacks. Hence, determining these biases and how they could be leveraged for malicious personalization is key for maximizing the success and efficiency of an attack. In general, self-disclosure biases have been investigated extensively in psychology through the lens of different theories and behavioral frameworks (Ellison et al., 2007; Steinfield et al., 2008; Stutzman et al., 2011). For instance, studies based on the *use and gratification theory* (McGuire, 1974) have focus on identifying adoption patterns among users of SNSs. That is, they analyse the psychological benefits of engaging in these platforms and sharing information across them (Min and Kim, 2015). In sum, these studies suggest that intrinsic factors like self-promotion (Mehdizadeh, 2010), impression management (Krämer and Winter, 2008), and social capital (Steinfield et al., 2008) may affect users' online behavior. Furthermore, factors like altruism (e.g., provide useful information to help friends) and group joy (e.g., exchange information while interacting in networked games) were also shown to influence people's information-sharing decisions in SNSs (Fu et al., 2017).

Other studies have focused on explaining people's information-sharing behavior through the lens of the *privacy-calculus* (Li et al., 2010; Dienlin and Metzger, 2016; Trepte et al., 2017). That is, they examine how people assess and weigh the costs and benefits of revealing private information when interacting in SNSs. Under this framework, people are expected to open their privacy boundaries (i.e., share more information about themselves) if they outweigh the expected benefits of sharing personal information over their privacy concerns (Laufer and Wolfe, 1977; Culnan and Armstrong, 1999). However, it has been shown that users not always enumerate and evaluate all these costs and benefits in a rational and objective way (Min and Kim, 2015; Trepte et al., 2017). Moreover, it is sometimes hard for regular users to anticipate the consequences of their information-sharing actions, and therefore to make sound privacy decisions (Wang et al., 2011). Hence, factors, such as low levels of literacy and privacy awareness can lead users to disclose information in SNSs which they later regret.

In addition to individual predispositions and cognitive biases, research has also addressed the role of the social context in people's information-sharing behavior (Acquisti and Gross, 2006; Lewis et al., 2008; Cheung et al., 2015; Choi et al., 2018). Overall, this view posits that people often behave in what they believe to be socially accepted ways in order to gain certain benefits so as to avoid social punishment or disapproval. Such socially-compliant decisions are normally made when users lack objective means to evaluate their own behavior (Cialdini and Goldstein, 2004). Social influence has been shown to be a critical factor that determines not only people's engagement in SNSs, but also their privacy behavior within these platforms (Cheung et al., 2015). Particularly, studies have shown that users tend to disclose information about themselves to comply with their peers' expectations (Cheung et al., 2015). Furthermore, they sometimes engage in self-disclosure activities to avoid isolation and, in some cases, to reduce the chances of being stigmatized by others. This last one has been observed in dating apps like *Grindr* in which users include their HIV status as part of their profile to increase their chances of finding a partner (Warner et al., 2018).

## 4. Method

All in all, user's information-sharing behavior is often influenced by their individual motivations and cognitive biases. Likewise, such a behavior can be fostered and guided through personalized incentive mechanisms embedded in the design of information systems. These incentives, when used by deceivers, can be seen as a case of *malicious personalization* in which users are misguided to disclose their private information to others with harmful intentions. In order to understand which cognitive and motivational biases are likely to be exploited by deceivers in SNSs, we have elaborated an online survey about people's willingness to share personal data under different incentives. In this section, the design of such survey is introduced together with the sampling approach.

### 4.1. Survey Design

To investigate the role of self-disclosure biases in malicious personalization we followed a scenario-based approach. Particularly, participants were asked to indicate their willingness to share pieces of private information under different scenarios. Each scenario represented a situation in which information is asked for apparently harmless purposes (like in deceptive attacks). In total 8 scenarios were included, one for each of the following information categories:

- *Identity*: comprises of identifying information about the users (e.g., name and address).
- *Social network*: covers information about the social circle and shared content (e.g., friends list and posts).
- *Health*: includes physical and health related information (e.g., physical condition).
- *Finances*: encompasses income/expenses and other financial information (e.g., credit card).
- *Education and occupation*: contains information that essentially forms an online résumé (e.g., education level and work experience).
- *Beliefs*: covers various personal beliefs and points of view (e.g., political and religious views).
- *Travels*: consists of information about visited locations (e.g., trips to cities and landmarks).
- *Geolocation*: includes geolocation data (e.g., travels and current GPS position).

For instance, the following scenario was elaborated for the “health” category:

“*You start using a fitness tracker/wearable to improve your jogging workout and control your performance. The device app wishes to collect information including your frequent trails, pace, and burnt calories to elaborate a fitness routine for beginners and, thereby, encourage other people to start a healthy lifestyle”*

As already mentioned, cognitive and motivational biases may guide user's privacy decisions. On the other hand, deceivers often exploit such biases to manipulate and misguide their victims. Hence, we included for each scenario a set of statements related to the following biases:

- *Financial gain:* The disclosure of personal information is motivated by a cash-equivalent reward, such as money, gifts and discount vouchers (Taylor et al., 2009). This bias could be exploited through a spear-phishing email that says “*We are pleased to announce that employees have the right to get a 50% discount on all of our online products”* and redirects the victim to a phishing page that requests her organizational credentials (i.e., ID and password) to access the discount prize.
- *Personal gain:* The user is motivated to share personal information for a reward that has no cash-equivalent value (Taylor et al., 2009). Such a reward may consist of personalized assistance, customization or any other benefit prized by the user. This bias could be exploited using a spear-phishing email that says “*This is your last chance to get a free premium account at Netflix!”* and asking the organizational credentials of the victim as the required information for the registration.
- *Moral gain (altruism):* The user discloses private information to help others without the expectation of a (not) cash-equivalent reward (Ma and Chan, 2014). For instance, achieving a sense of satisfaction after supporting another user who suffers from the same health condition (Chung, 2014). A deceiver may take advantage of this bias by impersonating a member of a prominent NGO through a fake account and asking to sign a fake petition related to a humanitarian cause.
- *Social compliance:* The users' privacy decisions are influenced by their social context (Cialdini and Goldstein, 2004). Thus, they are more willing to disclose personal information if members of their social circle are already doing it. A deceiver may exploit this bias by asking the victim to answer a fake survey or accessing a non-secure link on behalf of the victim's friends, family or acquaintances.
- *Unawareness:* The user is not able to foresee the (potential) negative consequences of sharing personal information. Hence, the benefits of disclosing such information outweigh the user's underestimated costs (Wang et al., 2011). A deceiver may exploit this bias by claiming to be working in the same company as the victim (e.g., in the IT department) and asking her to start putting confidential information in a non-secure cloud system.
- *Apathy:* The user perceives privacy violations as inevitable and control over personal data as already lost (Hargittai and Marwick, 2016). Such a feeling of resignation drives the user to outweigh the costs of sharing personal information over its potential benefits. A mobile app containing Malware could exploit this bias by simply asking the user to grant full permissions over the phone's GPS location or its photo gallery.

For instance, the *personal gain* statement for the “health” scenario was defined as “*If this would grant me access to premium features of the app, then I would allow the app to collect this information,”* and the corresponding *financial gain* statement as “*If on exchange I would get a voucher for buying sport clothes, then I would share this data.”* To evaluate participants' willingness to disclose personal formation, we asked them to indicate to which extent they agree with each of these statements (Table 1). For this, a 6-point Likert scale was used were 1 corresponds to “strongly disagree” and 6 to “strongly agree.”

**Table 1:** Self-disclosure biases defined for the “health” scenario.

**Self-disclosure bias**	**Survey statement**
Financial gain	“*If on exchange I would get a voucher for buying sport clothes, then I would share this data”*
Personal gain	“*If this would grant me access to premium features of the app, then I would allow the app to collect this information”*
Moral gain	“*Since this can help others to develop healthy habits, I would share this information without anything on exchange”*
Social compliance	“*I would share this information with the device if other users start contributing”*
Unawareness	“*I am fine with sharing this information since it is usually collected in an anonymous way”*
Apathy	“*I would give access to this information since these devices are already collecting it for other purposes anyway”*

Prior to the assessment of the scenarios, participants were asked to answer some questions about their usage of SNSs. Particularly, they were asked (i) how much time do they spend in these platforms, (ii) if they inform themselves about the privacy policies of SNSs, and (iii) if their profile information is made public to others. Participants were also asked to indicate their willingness to sell their private information to SNSs and the value they would assign to different data types. In particular, how cheap/expensive they would sell the information involved in the scenarios they had to evaluate afterwards (i.e., identity, social network, health, finances, education and occupation, beliefs, travels, and geolocation). Specifically, users rated each information category using a 6-point Likert scale where 1 corresponds to “very cheap” and 6 to “very expensive.”

### 4.2. Population and Sampling

The survey was conducted in August of 2019 through Amazon's Mechanical Turk^2^ (Mturk), a crowdsourcing marketplace where *requesters* can allocate Human-Intelligence Tasks (HITs) to be completed by the platform's *workers* (Paolacci et al., 2010). Mturk has become a popular platform for researchers to conduct experiments with human subjects particularly in the areas of usable privacy and security (Kelley, 2010). Our HIT was the survey described in section 4.1 and workers were required to have a HIT approval rate ≥95% and a number of approved HITs ≥ 1,000, as it is recommended for this type of task^3^. A remuneration of $1.25 was offered to each worker/participant considering an average completion time of 18 min per survey and the payment standards of the Mturk community. A total of 349 responses from participants of the United States and Canada was considered for the analysis and three were rejected. Table 2 shows the self-reported demographic characteristics of the study sample.

**Table 2:** Demographic characteristics of the studied sample.

**Demographic**	**Ranges**	**Frequency**	**Responses (%)**
Age	18–25 years	13	3.7
	26–35 years	149	42.7
	36–45 years	107	30.7
	46–55 years	46	13.2
	<56 years	34	9.7
Gender	Male	183	52.4
	Female	163	46.7
	Prefer not say	2	0.6
	Non-binary	1	0.3
Occupation	Employed full time	233	66.8
	Employed part time	27	7.7
	Home maker	13	3.7
	Retired	8	2.3
	Self employed	51	14.6
	Student	5	1.4
	Unable to work	4	1.1
	Unemployed	8	2.3
Education	Associate degree	45	12.9
	Bachelor degree	148	42.4
	Doctorate	4	1.1
	High school degree	37	10.6
	Less than high school	2	0.6
	Master degree	42	12
	Professional degree	6	1.7
	Some college, no degree	65	18.6

## 5. Results and Findings

Following, we summarize the results of our online survey^4^. Particularly, we analyse how users assess the value of particular pieces of personal information and compare it against their willingness to disclose them under the influence of cognitive and motivational biases (as described in section 4.1). For this, descriptive metrics were elaborated to identify the most reported biases for each scenario. Moreover, a correlation analysis was conducted to investigate relations between survey items. Particularly, to identify correlations between people's willingness to share their personal data and the value they assign to them.

### 5.1. Cognitive and Motivational Biases

**Figure 1:**
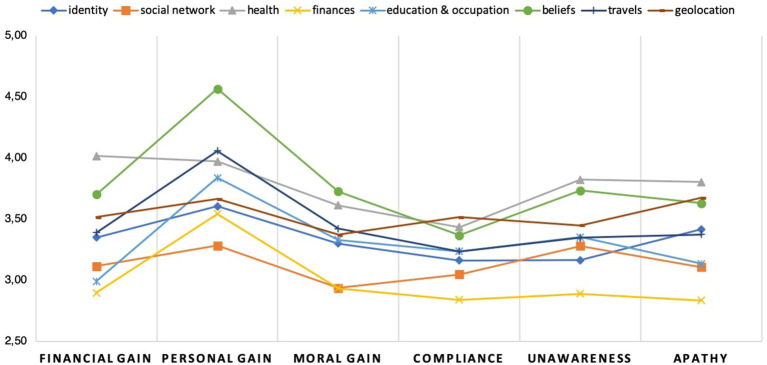
Users' reported cognitive and motivational biases for each scenario (item average).

**Figure 2:**
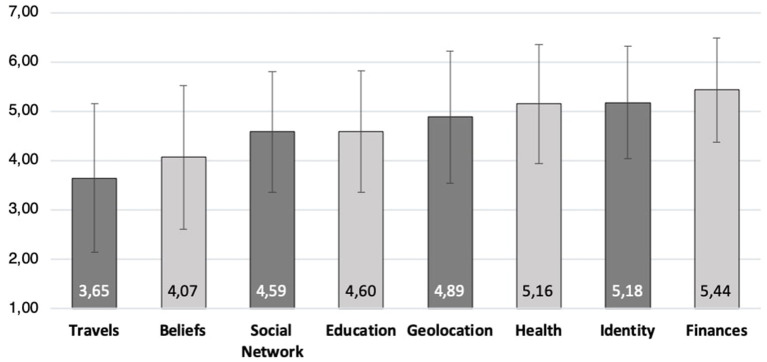
User's reported value for each data type (item average).

Figure 1 summarizes the participants' assessment of the proposed scenarios. Particularly, their average willingness to share personal data on each specific scenario. As already mentioned, a scenario involves specific type of information and proposes a set of statements related to cognitive and motivational self-disclosure biases. For instance, one can observe that *compliance* and *apathy* are the weakest biases in the scenario concerning financial information. Moreover, together with *moral gain, financial gain*, and *unawareness*, have the lowest score across all the scenarios. As Figure 2 illustrates, the average value assigned to financial data is the highest of all (M = 5.18 ± 1.139). Hence, this proposes (in principle) that information of high value is less likely to be shared by the users in the context of a deceptive attack. However, reported intentions of sharing other highly-valuable data types like health (M = 5.16 ± 1.211) and identity (M = 5.18 ± 1.139) is high in comparison to other information categories. Furthermore, the statements corresponding to *unawareness* and *apathy* have their highest values on the “health” scenario.

Among all the biases, *personal gain* has its highest peak in the “beliefs” scenario and its second highest in the one of “travels.” Moreover, as shown in Figure 2, the data corresponding to “beliefs” together with the one of “travels” were reported by the participants as the ones with the lowest value (beliefs: M = 4.07 ± 1.454; travels: M = 3.65 ± 1.51). This suggests, in principle, that *personal gain* can be an influential factor when users are asked for data with a relative low value. However, *personal gain* was also the bias with the highest average score within the “finances” scenario being financial information the one with the highest value. Moreover, this is also the case for the scenarios corresponding to “identity,” “social network,” “occupation and education,” and “travels.” Hence, *personal gain* seems to be, in general, the strongest motivation across all the proposed scenarios with the exception of “geolocation” and “health” whose peak correspond to *financial gain* and *apathy*, respectively. On the other hand, *compliance* was the bias with the lowest average score except for the scenarios corresponding to “geolocation” and “social network” in which *moral gain* was rated as the lowest. Likewise, *financial gain* was the bias with the lowest average score in the “education and occupation” scenario.

### 5.2. Willingness to Share Data

To further investigate users' cognitive and motivational biases when disclosing personal information, we ran an ordinal logistic regression (Table 3) which is a widely used method for analysing correlations between Likert items (O'Connell, 2006). For this, the willingness to disclose personal information was defined as the dependent variable and the value of such information as the predictor (“data value”). Therefore, for the eight scenarios/data-types and the six self-disclosure biases, a total of 48 regression analysis were conducted. In addition, the survey items corresponding to (a) having a public profile (“public profile”), and (b) being aware of the privacy policies of SNSs (“policy-aware”) were used as control variables.

**Table 3:** Logistic regression results: ordered log-odds (B) of disclosing personal data on a deceptive scenario.

**Bias**		**Identity**	**Social network**	**Health**	**Geolocation**	**Travels**	**Beliefs**	**Education**	**Finances**
Financial gain	Data value	−0.390^***^	−0.237^*^	−0.139^†^	−0.186^*^	−0.159^*^	−0, 150^*^	−0.093	0.006
	Public profile	0.548^***^	0.537^***^	0.334^***^	0.510^***^	0.553^***^	0.409^***^	0.501^***^	0.235^***^
	Policy-aware	0.085	−0.005	−0.054	0.163^*^	0.235^*^	0.123	0.071	0.127^†^
Personal gain	Data value	−0.254^*^	−0.227^*^	−0.086	−0.113	−0.224^***^	−0.288^***^	−0.207^**^	0.026
	Public profile	0.317^***^	0.520^***^	0.372^***^	0.339^***^	0.296^***^	0.065	0.194^***^	0.182^*^
	Policy-aware	0.021	−0.042	−0.090	0.019	0.052	−0.081	−0.021	0.063
Moral gain	Data value	−0.321^***^	−0.187^*^	−0.228^***^	−0.140^†^	−0.218^***^	−0.158^*^	−0.116	−0.082
	Public profile	0.524^***^	0.546^***^	0.360^***^	0.501^***^	0.0439^***^	0.410^***^	0.421^***^	0.269^***^
	Policy-aware	0.088	0.116	−0.009	0.144^†^	0.194^*^	0.139^†^	0.063	0.025
Social compliance	Data value	−0.214^*^	−0.168^*^	−0.210^**^	−0.158^*^	−0.179^†^	−0.191^**^	−0.122	0.125
	Public profile	0.546^***^	0.501^***^	0.426^***^	0.486^***^	0.481^***^	0.527^***^	0.459^***^	0.201^***^
	Policy-aware	0.098	0.041	0.121	0.112	0.210^†^	0.201^**^	0.093	0.098
Unawareness	Data value	−0.332^***^	−0.203^**^	−0.081	−0.173^*^	−0.186^**^	−0.208^**^	−0.082	−0.045
	Public profile	0.432^***^	0.368^***^	0.431^***^	0.508^***^	0.375^***^	0.301^***^	0.424^***^	0.301^***^
	Policy-aware	−0.071	0.053	0.01	0.196^*^	0.042	−0.003	−0.011	0.038
Apathy	Data value	−0.275^**^	−0.262^***^	−0.186^*^	−0.091	−0.156^*^	−0.201^**^	−0.102	−0.104
	Public profile	0.537^***^	0.468^***^	0.404^***^	0.453^***^	0.490^***^	0.439^***^	0.479^***^	0.241^***^
	Policy-aware	0.065	0.033	0.049	0.094	0.119	0.125	0.117	0.065

† 0.05 < p ≤ 0.10;

* p ≤ 0.05;

** p ≤ 0.01;

*** *p ≤ 0.001; β = 95%*.

Table 3 shows the ordered log-odds (B) of the predictors for each bias and disclosure scenario. For instance, one can observe that the log-odds for the reported value of “identity” data is B=-0.390 when the bias is *financial gain*. This means that, for this particular bias, the likelihood of disclosing identity data decreases around |(*e*^−0.39^ − 1) * 100| = 32.29% as its value (i.e., the value assigned to “identity” data) increases in one unit. Likewise, this likelihood increases around |(*e*^0.548^ − 1) * 100| = 72.98% for those who reported having a public SNS profile. However, there is no statistical significance in relation to the participant's extent of awareness on SNSs' privacy policies.

In general, we observe that, independently of the data type and self-disclosure bias, there is no statistical significance between participants' policy awareness and their reported willingness to disclose personal information. However, having a public SNS profile has shown to have a connection with the reported self-disclosure motivations and cognitive biases. For instance, for biases like *financial* and *moral gain*, the likelihood of disclosing identity, social network and geolocation data increases more than 65% as the survey item “public profile” increases in one unit. This is also the case of *personal gain* and *unawareness* for information related to social network and geolocation, respectively. Furthermore, for *apathy*, the chances of revealing data related to education, identity, and travels rise about 60% per unit of increase in “public profile.” Nevertheless, this probability goes bellow 35% in the case of financial information for all the biases. This in principle could be related to the high value assigned to this type of information. However, our sample lacks statistical significance to support this hypothesis. Moreover, “data value” has, in general, very low statistical significance or B-values across the different scenarios and self-disclosure biases.

## 6. Discussion

Overall, the results of our survey show that self-disclosure biases can vary when people are asked to reveal particular data types. Moreover, a correlation was observed between participants' willingness to reveal personal data and having a public SNSs profile. However, we could not identify correlations for the value participants assign to particular pieces of information, nor their reported awareness level on privacy policies. In the following subsections we discuss the limitations of our approach and elaborate a set of countermeasures based in our findings. The purpose of such countermeasures is to raise awareness among the users of SNSs regarding the potential consequences of revealing private information to deceivers.

### 6.1. Countermeasures

In order to elaborate deception countermeasures, we first analyse current state-of-the art approaches. Hence, methods and techniques for detecting fake accounts and deceptive messages are discussed in section 6.1.1, and countermeasures are introduced in section 6.1.2. Particularly, the latter section highlights how the findings presented in section 5 can be utilized for the development of personalized risk awareness mechanisms which combine existing approaches together with persuasive technologies.

#### 6.1.1. Current Approaches

Scholars have introduced different strategies to identify deceptive messages and fake accounts in SNSs (Briscoe et al., 2014; Alowibdi et al., 2015; Mulamba et al., 2018; van der Walt et al., 2018). For instance, Briscoe et al. (2014) developed a machine learning model that can detect if a text message sent over a SNS communication channel (e.g., post, tweet, or instant message) is truthful or deceptive. For this, the model uses linguistic cues like the average sentence length, complexity, and sentiment as predictors of deception. On the other hand, Alowibdi et al. (2015) developed a classifier capable to identify inconsistencies in Twitter profiles based on a set of deception indicators (e.g., profile layout colors, first name, and user-name). Particularly, such classifier can detect gender or location inconsistencies in a profile and, thereby, classify its corresponding account as fake. In line with this, van der Walt et al. (2018) followed a similar approach to flag deceptive accounts but using additional predictors, such as tweets geo-tags, name length, and friends/followers ratio.

Detecting deceptive accounts and messages is a first attempt on safeguarding the users from harmful online experiences. Furthermore, it is a major step toward ensuring safer interactions through SNSs. However, attacks are getting more sophisticated and, as we can see from the results of our survey, people can be misled to reveal personal information when incentives and motivational biases outweigh their privacy concerns. This demands more effective awareness tools as these instruments play a key role in supporting users when making online privacy decisions. For instance, D́ıaz Ferreyra et al. (2019) propose the use of *risk patterns* to alert users when they are about to disclose private information inside social media posts. However, to the best of our knowledge, not many efforts have been made on informing the users about the risks of disclosing personal information to deceivers. Particularly, on developing technologies that alert users when they are about to reveal personal data to an attacker.

#### 6.1.2. Personalized Risk Awareness

Overall, current advances on privacy awareness can provide a suitable framework for developing countermeasures against online deception (Petkos et al., 2015; Díaz Ferreyra et al., 2017; De and Le Métayer, 2018). For example, using risk patterns similar to the ones introduced by Díaz Ferreyra et al. (2017) one could define the pre- and post-conditions of a deceptive scenario as a triple <*PI, Deceiver, UIN>* where *PI* corresponds to private information, *Deceiver* to a set of deception queues, and *UIN* to an unwanted incident. Under this representation, the unwanted incident *UIN* corresponds to the *post-condition* of a deceptive attack and revealing the information *PI* to a user with *Deceptive* characteristics to the *pre-condition*. This would allow us, for instance, to represent a scenario in which identity theft (*UIN*) occurs after a user reveals her user-name and password (*PI*) to another user whose account has been flagged as potentially deceptive (*Deceiver*). Furthermore, a collection of well-known deceptive scenarios expressed in this format could serve the generation of warning messages when the *pre-condition* of one or more patterns is satisfied. For example, showing a pop-up message like “*It seems you are about to reveal <PI>to a user who may be a deceiver. This could derive in a case of <UIN>”* and replacing the place-holders <*PI>* and <*UIN>* with the values defined in the corresponding pattern. This strategy is similar to the one employed by Intelligent Tutoring Systems which are used in learning environments to provide personalized instructional content to students (D́ıaz Ferreyra, 2019).

The use of interventions (i.e., warning messages or suggestions) is a promising approach for nudging users' privacy behavior (Acquisti et al., 2017). However, it has also been shown that such interventions may result annoying for users with low privacy concerns (Wang et al., 2013). Hence, warnings should be aligned somehow with the privacy goals and expectations of each individual user. In other words, privacy-awareness mechanisms should incorporate adaptivity principles into their design to better engage with their users (D́ıaz Ferreyra et al., 2019). One of the findings that could contribute in the design of adaptive awareness mechanisms is the one related to the users' profile visibility. Particularly, the *frequency* and *content* of interventions could be tailored using the visibility of the user's profile as an adaptation variable. Moreover, it could be used in combination with the users' privacy attitudes (Ghazinour et al., 2013), risk aversion (D́ıaz Ferreyra et al., 2019), and digital literacy (Wisniewski et al., 2017) which have already been proposed as variables of adaptation.

On the other hand, the results of our survey also suggest that the influence of self-disclosure biases may vary among users of SNSs. That is, whereas a particular bias can drive a user to disclose her data to a deceiver, the same bias may not influence the behavior of another user under a deceptive attack. Hence, different privacy-awareness strategies may be necessary to deal with the effects of different self-disclosure biases. This could be done, for instance, by framing the *style* of the interventions according to the bias they are addressing (Kaptein et al., 2012). Particularly, interventions may adopt a more *authoritarian* style (e.g., “*Rethink what you are going to provide. Privacy researchers from Harvard University identify such information as highly sensitive!”*) or a more *consensual* one (e.g., “*Everybody agrees: Providing sensitive information can result in privacy risks!”*) depending on the bias they try to counteract (Schäwel and Krämer, 2018). For instance, for users whose more salient bias is *personal gain*, a more authoritarian style could persuade them better than a consensual one. Conversely, for those motivated mainly by *social compliance*, a consensual style may be the most adequate. Besides, warnings could incorporate additional information related to privacy protection mechanisms (e.g., how to block or report a user) to counteract the effect of *apathy*. Furthermore, interventions could also provide links to relevant news and media articles about deception to target *unawareness* or *moral gain* (De and Le Métayer, 2018). In the case of *financial gain*, incorporating information about the value of data together with reputation queues of the data requester may be a good strategy to promote a safer privacy behavior.

### 6.2. Limitations

Although the approach employed in this work has yielded interesting results, there are some limitations that should be acknowledged. First of all, our results are based on hypothetical self-disclosure scenarios which were evaluated by the participants of our survey. This approach does not ensure that, in a real case scenario, their behavior would be consistent with what they have reported. Likewise, the statements corresponding to the cognitive and motivational biases we defined should be elaborated further, especially in the form of validated Likert scales. On the other hand, using Mturk for conducting online surveys supposes a loss of control over the experimental setting on a large extent (Kittur et al., 2008; Paolacci et al., 2010). In particular, participants may get distracted in their physical environment and, thereby, compromise the quality of their answers. Furthermore, workers sometimes provide fast or nonsense answers in order to make more money in less time. Nevertheless, it has been shown that the Mturk platform can provide results as relevant as those from traditional survey methods (Paolacci et al., 2010). This can be achieved by applying a number of good practices, such as controlling the time workers actually spend in the task or filter out workers with a low HIT approval rate (Amazon, 2011; Oh and Wang, 2012). Such practices were followed to ensure good quality results.

## 7. Conclusions and Future Work

Safeguarding people's private information is extremely important for the welfare of modern societies. However, increasing the security levels around such information is not enough since nowadays it is possible to monitor and analyse people through their SNS profiles. This makes cyber-attacks very easy to personalize according to what hackers may find about their victims in these online platforms. It is not a secret that, for example, identity theft affects millions of people a year costing victims countless hours and money in identity recovery and repair. The much-publicized Equifax scandal that broke out in September 2017—after the personal information of as many as 143 million Americans had been compromised (and an untold number of Canadians and Brits)—has resulted in the recent resignation of the Equifax CEO. Even Hollywood makes films about cases of extreme lack of privacy, such as *The Circle*, and about personalization of phishing attacks, such as *CSI: Cyber*.

In sum, we need to provide a better future for the next generation of Internet users since it will be born in an age in which privacy may appear as an anomaly. However, people will remain susceptible to manipulation and privacy risks unless coordinated actions between developers of media technologies, users, government, and the civil society are jointly taken. This work has explored the exploitable biases for malicious personalization in SNSs and elaborated countermeasures which incorporate current advances in risk awareness, personalization and persuasive technologies. We believe that such countermeasures are a promising approach for engaging users of SNSs (specially teenagers) in a sustained privacy-learning process. Moreover, the premise of such countermeasures is not banning people from sharing status updates, photos and networking, but to support them in their individual privacy decisions. This would not only increase their levels of risk awareness but also allow them to disclose private information at their own responsibility.

As mentioned throughout this work, deceptive attacks are hard to identify since deceivers employ different strategies (i.e., motivations and incentives) to influence and mislead their victims. Moreover, such attacks can be crafted and personalized to the particular self-disclosure biases of the targeted victim in order to maximize their damage. Hence, understanding the cognitive and motivational biases exploited by deceivers is necessary for shaping privacy-preserving technologies to protect the users. The results of this work suggest that, in principle, the effect of each bias vary from individual to individual. Therefore, technical countermeasures as well as training and awareness programs should be personalized according to the biases that are more exploitable for each particular user. Moreover, the use of risk communication strategies is a promising approach for designing personalized countermeasures and will be investigated in further publications.

One of the most salient findings of this work is the relation between users' profile visibility and their willingness to share private information under a deceptive attack. Specifically, it was observed that participants who reported having a public profile were more willing to disclose personal data in a deceptive scenario. Therefore, profile visibility is proposed as a potentially significant adaptation variable for deception countermeasures. However, recent research in online self-disclosure has found no differences in the self-disclosure practices of users with a public SNS profile and those with a private one (Gruzd and Hernández-Garćıa, 2018). Nevertheless, that study did not take into consideration the influence that incentive mechanisms together with cognitive and motivational biases may have on users' privacy practices. Hence, we intend to research this point in more detail, in order to further corroborate our results.

Another aspect that should be analyzed in more detail are the cultural factors that may influence people's privacy decisions. Particularly, the results of this work are based on a sample consisting of Americans and Canadians which, according to the Hoftede's taxonomy, are *individualistic* societies (Li et al., 2017). That is, they tend to care more of themselves and their inner circle, and exhibit a behavior which is mainly driven by individual achievements. Conversely, in *collectivist* societies, such as Mexico or Spain, people often reflect on the consequences that their actions may have on others; particularly on the members of their social context (e.g., extended families, clans, or organizations) (Hofstede, 2011). Thus, some of the results presented in this work may be closely connected to the cultural background of the survey participants. For instance, the prevalence of “personal gain” in most of the scenarios may be due to the individualistic nature of the sample among other cultural factors. Hence, future research will investigate further the effects of the social context on the motivations and cognitive biases which are frequently exploited by deceptive attackers.

## Data Availability Statement

All datasets generated for this study are included in the article/Supplementary Material.

## Ethics Statement

The studies involving human participants were reviewed and approved by The Ethics Committee of the University of Montreal. The patients/participants provided their written informed consent to participate in this study.

## Author Contributions

The study was outlined and conceived by EA and HH. ND organized the dataset, performed the statistical analysis and wrote the first draft of the manuscript. All authors contributed to the design of the study, manuscript revision, read, and approved the submitted version.

### Conflict of Interest

The authors declare that the research was conducted in the absence of any commercial or financial relationships that could be construed as a potential conflict of interest.

## Footnotes

^1^Estimates of the number of Social Engineering based cyber-attacks into private or government organizations—https://bit.ly/2k5VKmP (accessed 07/09/2019).

^2^Mturk —www.mturk.com

^3^Tips for Academic Requesters on Mturk—http://turkrequesters.blogspot.com/2012/09/tips-for-academic-requesters-on-mturk.html (accessed 07/09/2019).

^4^Survey data is available as Supplementary Material.
